# Clinical and Perioperative Determinants of Postoperative Pneumonia After Craniotomy for Tumor Resection

**DOI:** 10.3390/jcm15124437

**Published:** 2026-06-08

**Authors:** Anatoli Pinchuk, Nikolay Tonchev, Anna Schaufler, Claudia A. Dumitru, Belal Neyazi, Klaus-Peter Stein, I. Erol Sandalcioglu, Ali Rashidi

**Affiliations:** Department of Neurosurgery, Otto-von-Guericke-University Magdeburg, 39120 Magdeburg, Germany; anatoli.pinchuk@med.ovgu.de (A.P.); nikolay.tonchev@med.ovgu.de (N.T.); anna.schaufler@med.ovgu.de (A.S.); claudia.dumitru@med.ovgu.de (C.A.D.); belal.neyazi@med.ovgu.de (B.N.); klaus-peter.stein@med.ovgu.de (K.-P.S.); erol.sandalcioglu@med.ovgu.de (I.E.S.)

**Keywords:** craniotomy, postoperative pneumonia, brain tumor, risk factor

## Abstract

**Background/Objectives**: Postoperative pneumonia is a common complication in surgical patients. Despite its clinical significance, there is limited evidence regarding its occurrence following intracranial tumor resection, the most common procedure in neurosurgery. The objective of this study is to determine the incidence of postoperative pneumonia, to examine its association with length of hospital stay, and to identify potential risk factors. **Methods**: A retrospective cohort study was conducted on 1481 patients who underwent intracranial tumor resection in our department over a ten-year period, excluding the influence of anticoagulant or antiplatelet medications. **Results**: Of the 1481 patients included in this study, postoperative pneumonia occurred in 1.48% of cases. Smoking status (*p* = 0.014) and prolonged hospital stay (*p* = 0.011) emerged as significant risk factors in the univariate analysis for postoperative pneumonia in patients undergoing brain tumor resection. In contrast, demographic factors (age, sex, body mass index), pre-existing comorbidities (hypertension, diabetes, cardiovascular disease, chronic inflammatory conditions), and laboratory parameters did not show significant associations with the development of postoperative pulmonary infection. **Conclusions**: This study identified pre- and postoperative risk factors associated with pneumonia following craniotomy for intracranial tumors. These findings may provide a valuable framework for pre- und postoperative risk assessment and guide strategies to mitigate the occurrence of postoperative pneumonia.

## 1. Introduction

It is reported that each year, approximately 22.6 million people experience neurological conditions or injuries that necessitate neurosurgical expertise, and of these, approximately 13.8 million require surgical treatment [1]. Postoperative pneumonia ranks as the third most common complication following surgery and is associated with poor prognosis and substantial healthcare costs [2,3,4,5,6]. As one of the most common complications in neurosurgery, postoperative pneumonia not only prolongs hospital stays but also increases the risk of postoperative mortality [3,4,5,6,7]. Nevertheless, this clinical complication of surgical therapy is frequently underestimated and not considered during the evaluation of general perioperative risk and pre-surgical patient consultations.

Several risk factors, including advanced age, diabetes, and chronic obstructive pulmonary disease, have been linked to the development of postoperative pneumonia in both general and cardiothoracic surgery [6,8,9].

Previous studies have identified a range of patient-specific and surgical factors that are strongly associated with the development of postoperative pneumonia, including preoperative anemia, hypoalbuminemia, tumor type, American Society of Anesthesiologists classification, smoking history, duration of surgery, and use of mechanical ventilation [6,10,11,12].

However, many of these factors are challenging to modify or optimize prior to surgery. Most studies characterize postoperative pneumonia as the occurrence of atelectasis, pulmonary edema, exacerbation of pre-existing chronic lung disease, or respiratory failure in patients following surgery [13,14]. The presence of these differences in nomenclature is driven mostly by the differences in healthcare systems between different countries and economic regions. This may also be the rationale behind the decision to perform such complex surgical procedures in specialized medical centers that possess the capacity to not only address the pathological condition, but also to manage any potential complications that may arise during the course of treatment.

Neurosurgical procedures are considered particularly high risk for this complication [15]. A plausible explanation for the elevated risk is that craniotomy can lead to reductions in lung volume and arterial blood gas tensions, accompanied by alterations in respiratory patterns [16]. Furthermore, specific neurological deficits predispose patients to immobilization, which in turn can lead to the development of postoperative pulmonary infections.

Despite its clinical significance, little is known about how often postoperative pneumonia occurs or how it affects outcomes following craniotomy, the most commonly performed procedure in neurosurgery. Gaining a clearer understanding of its incidence, risk factors, and potential impact on patient outcomes is crucial for guiding targeted strategies to prevent this complication. This study seeks to clarify how often postoperative pneumonia occurs, explore its relationship with the duration of hospital stay, and identify the factors that may place patients at higher risk.

## 2. Materials and Methods

This retrospective, single-center study was completed in the Department of Neurosurgery at University of Magdeburg, covering a ten-year period from 2008 to 2018. The data was derived by a medical specialist to reduce the possibility of misinterpretation of the medical results and postoperative treatment of patients.

A total of 1480 consecutive adult patients who underwent elective craniotomy for intracranial tumor resection with general anesthesia were included. The study included a range of both benign and malignant entities, with a particular prevalence of meningioma, glioblastoma, pituitary adenoma and cranial metastases. Patients who received perioperative anticoagulants or antiplatelet agents were excluded to eliminate any potential influence from postoperative hemorrhage and associated reoperations, prolonged hospital stays, and an increased risk of pneumonia. Additional exclusion criteria included pre-existing cranial wound infection and incomplete clinical data.

The data presented in this study was obtained from electronic medical records and operative reports. The following variables were collected: demographic characteristics (age, sex, blood type, smoking history), pre-existing comorbidities (hypertension, diabetes mellitus, cardiovascular disease, chronic kidney disease, chronic inflammatory conditions, chronic liver disease), and preoperative laboratory values (platelet count, partial thromboplastin time [PTT], prothrombin time, C-reactive protein, white blood cell count). Furthermore, a range of surgical parameters were documented, including the duration of surgery, the surgical approach, and intraoperative findings.

The primary outcome was the occurrence of postoperative pneumonia within 30 days of surgery. Pneumonia was defined within the framework of nosocomial infection surveillance according to the following criteria: onset of pulmonary infection occurred no earlier than 48 h after surgery, and a new, persistent, or progressive infiltrate was identified on chest radiography. In addition, clinical symptoms were present, with at least two of the following criteria required: fever (>38.3 °C) or hypothermia (<36.5 °C), leukocytosis (>10,000/μL) or leukopenia (<4000/μL), purulent secretions from the lower respiratory tract or a change in the characteristics of respiratory secretions, and deterioration in gas exchange (e.g., hypoxemia or increased oxygen requirement) including microbiological confirmation and targeted antibiotic therapy.

### Statistical Analysis

The use of descriptive statistics was employed to summarize the baseline characteristics. Categorical variables were analyzed using either the chi-square test or Fisher’s exact test. In contrast, continuous variables were compared using Student’s *t*-test or the Mann–Whitney U test, depending on the distribution of the data. To identify risk factors for post-operative wound infections, a multivariate logistic regression model was constructed, with variables that had a *p*-value of less than 0.05 in the univariate analysis being entered into the model. The statistical significance was set at *p* < 0.05. The analyses were carried out using R version 4.5.1, incorporating the packages gtsummary version 2.3.0 for statistical testing and ggplot2 version 3.5.2, as well as gghalves version 0.1.4 for visualization.

## 3. Results

### Demographics of the Study Population and Risk Factors for Postoperative Pneumonia

**Figure 1:**
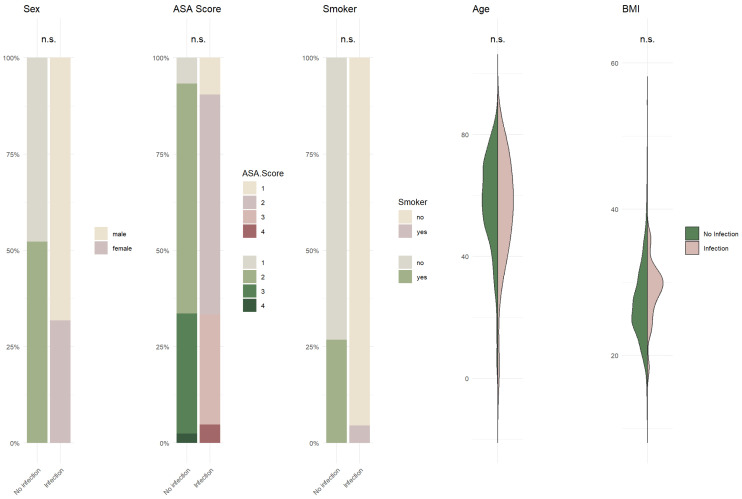
Distribution of the influence of various demographic parameters on wound healing after cranial surgery.

Twenty-two cases of postoperative pneumonia were identified from 1481 craniotomy procedures (intracranial tumor resection), an incidence rate of 1.48%. As shown in Table 1, several risk factors for postoperative pneumonia were identified, including the average age of patients with postoperative pneumonia (55 years; *p* = 0.5). Gender distribution did not play a significant role (*p* = 0.083). Of the 22 patients with pneumonia, 15 (68%) were male. However, other influencing factors such as Body-Mass-Index (BMI) and American Society of Anesthesiologists physical status classification (ASA-Classification) did not show a significant correlation (BMI *p* = 0.089 and ASA *p* = 0.6). A similar risk factor to smoking status (*p* = 0.014) was associated with postoperative pneumonia (Figure 1, Table 1).

**Figure 2:**
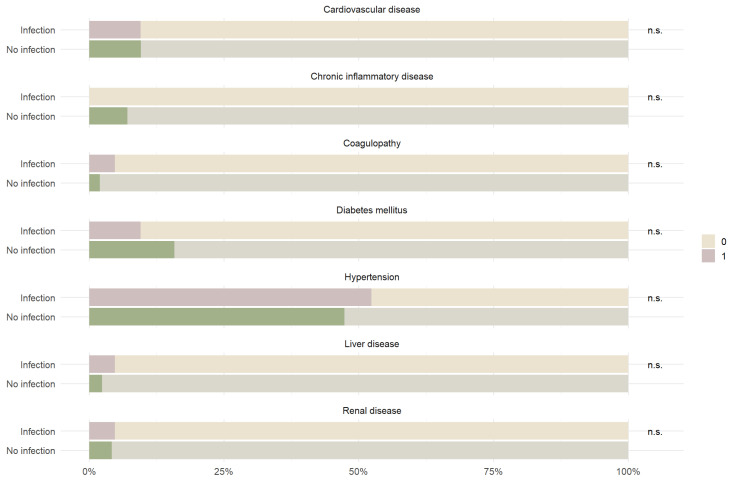
Graphic distribution of the influence of pre-existing conditions on postoperative pneumonia after cranial surgery.

Of 698 patients with arterial hypertension, 11 developed postoperative pneumonia (*p* = 0.8). Of 228 patients with diabetes mellitus, two developed postoperative lung infection (*p* = 0.6). Of 142 patients with coronary heart disease, two patients developed postoperative lung infection (*p* = 0.9). Chronic inflammation (*p* = 0.4) and liver disease (*p* = 0.4) also did not show evidence of a significant association (Figure 2, Table 1).

**Figure 3:**
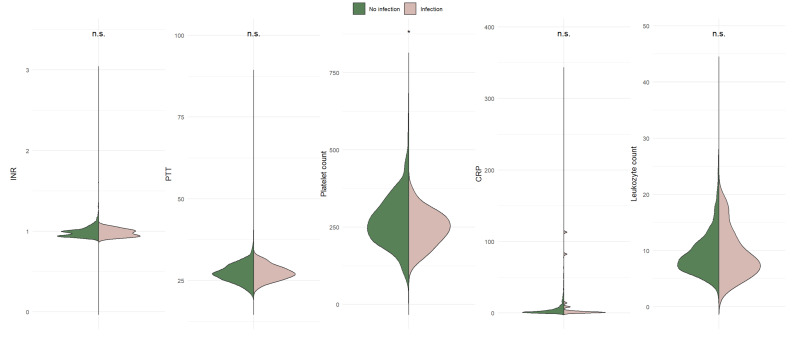
Graphic distribution of the influence of various laboratory parameters on postoperative pneumonia after cranial surgery.

Laboratory parameters such as CRP showed no evidence of a significant correlation with wound-healing disorders (*p* = 0.7). The platelet count (*p* = 0.047) showed a weak correlation. The complete blood count parameters, such as the leukocyte count (*p* = 0.8) and partial thromboplastin time (*p* = 0.9), also did not show significant correlations (Figure 3, Table 2). In the multivariate analysis, the duration of stay was significant. Smoking status showed only a trend but was not significant (Table 3).

**Figure 4:**
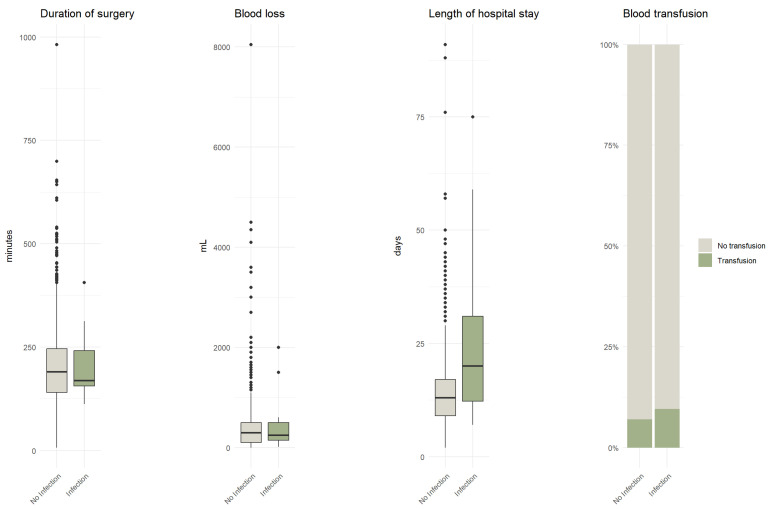
Graphic distribution of the influence of perioperative factors on postoperative pneumonia after cranial surgery.

In patients with postoperative pneumonia, the average duration of surgery was 201 ± 74 min (*p* = 0.9). Blood loss is an important factor, but no significant correlation (*p* = 0.8) between blood loss and pneumonia could be established. However, there was a significant correlation with length of stay (*p* = 0.011) (Figure 4, Table 1).

## 4. Discussion

Postoperative pneumonia is one of the most common complications in all surgical patients [3,5,6]. Postoperative pneumonia is associated with an unfavorable prognosis. Previous studies have demonstrated that pneumonia is linked to prolonged hospital stays and substantially increased hospital costs [7], which is consistent with the findings of the present study. Therefore, the early identification of risk factors for pneumonia is essential for targeted prevention and, consequently, for improving patient outcomes. Several studies have linked smoking status to an increased risk of pneumonia [17,18,19]. In our study, univariate analysis showed that smoking status was associated with an increased risk of pneumonia following surgery. In the multivariate analysis, smoking status showed a trend but was not significantly associated.

A low platelet count in some studies was associated with an increased risk of pneumonia [20,21]. In our study, the univariate analysis showed that the low platelet count was associated with an increased risk of pneumonia after surgery. However, this finding was not significant in the multivariate analysis. Additional attention should be directed to patients at elevated risk and preoperative platelet substitution therapy may be considered in high-risk patients.

This study, which analyzed a large regional database, found an incidence of pneumonia of 1.48% among patients undergoing craniotomy. This data aligns with the recently published results from a systematic review and meta-analysis on systemic complications following glioma resection [22]. The findings by K. Slychan et al. emphasize the clinical relevance of systemic complications on the primary outcome of neurooncological surgery patients. Age, sex, low body mass index, pre-existing conditions such as diabetes and hypertension, and routine laboratory parameters were not identified as risk factors for postoperative pneumonia after craniotomy in our cohort. However, pneumonia, defined as a pulmonary infection occurring at least 48 h after operation, was associated with a prolonged length of hospital stay. Among all postoperative complications, pneumonia had the second largest impact on prolonging the length of stay. Immobilization, worsening of neurological status and several other factors could be seen as additional reasons for a prolonged hospital stay. However, it is only through the strict definition of postoperative pneumonia that it is possible to determine whether this is a risk factor or an outcome.

It has been previously identified that patients over the age of 65 or 70 years old are more susceptible to developing pneumonia during the postoperative period following gastrectomy [23]. However, it is important to note that the elderly population is increasingly requiring surgical intervention, and age is becoming a less significant factor in the decision-making process for surgical treatment.

For instance, an earlier study reported that men were more likely to develop pneumonia after surgery for pituitary tumors [24]. In contrast, the present study did not identify age or male sex as risk factors for pneumonia.

Pneumonia occurs with markedly different frequencies depending on the underlying disease and surgical procedure, with reported rates of 0.6% in patients with pituitary tumors [24], 1.3–4.4% in patients with meningioma [25], 11.9% in patients with vestibular schwannoma [26], and 27.2% in patients undergoing microsurgical clipping of ruptured intracranial aneurysms [27]. These differences are not unexpected, as the underlying pathology can substantially affect the patient’s neurological status and thereby impair swallowing function or general alertness, which in turn increases the risk of pneumonia. Patients suffering from a deficit of the lower cranial nerves are predisposed to dysphagia and, in some cases, prolonged ventilation in the context of ICU therapy. The localization of the tumor within the cranial cavity may be an additional factor in the duration of the postoperative ventilation period. Extensive cranial base lesions together with markedly large supratentorial tumors frequently necessitate prolonged ventilation.

Additional attention should be drawn to the influence of general anesthesia on postoperative recovery and the risk of aspiration-related complications. The systematic review and meta-analysis by Natalini et al. [28] drew the conclusion that awake craniotomies in monitored anesthesia care could lead to a shorter surgical procedure time and length of stay. Differences in anesthetic approach may influence postoperative outcomes and should therefore be considered when interpreting complication rates across different surgical cohorts. Given the absence of data on awake craniotomies in the current study cohort, no conclusions can be drawn regarding this aspect.

A low BMI may reflect poor nutritional status and has been associated with impaired immune function and an increased risk of infectious diseases [29]. Bohl et al. reported that functional dependence is a risk factor for adverse outcomes after anterior cervical decompression and fusion [30]. In contrast, the present study did not identify body mass index as a risk factor for postoperative pneumonia. The present findings may be a consequence of two separate factors: firstly, the low number of obese patients in the present cohort, and secondly, the low incidence of postoperative pneumonia.

Another factor that has consistently been reported to be strongly associated with postoperative complications is the ASA-Classification. According to the American College of Physicians, an ASA physical status of class II or higher is considered a significant risk factor for postoperative pneumonia in patients undergoing non-cardiothoracic surgery [15]. In contrast, the present study did not find that patients with ASA class III had an increased predisposition to developing postoperative pneumonia.

The association between functional dependence and increased time spent in the supine position has been demonstrated to result in reduced vital capacity, impaired clearance of airway secretions and weakened cough effort [31]. After craniotomy, functionally dependent patients may remain in the supine position for a prolonged period, thereby increasing their risk of developing postoperative pneumonia.

Similarly, hypertension has been identified as a risk factor for postoperative pneumonia after cardiac surgery [32]. Hypertension is associated with overactivation of the sympathetic nervous system, which can lead to immunodeficiency, and with silent cerebral infarction, which is a predictor of aspiration pneumonia due to dysphagia [33]. Optimizing these factors may therefore offer an opportunity to prevent postoperative pneumonia. In the present study, however, hypertension was not identified as a distinct risk factor for pneumonia.

Prolonged operative duration was not associated with postoperative pneumonia in the present study. Strobel et al. reported that emergency status is a predictor of postoperative pneumonia after coronary artery bypass grafting [34]. Many emergency patients are unconscious or comatose due to severe cerebrovascular disease or brain tumors, which may predispose them to aspiration pneumonia. Detailed information on pneumonia subtypes, such as bacterial or viral pneumonia, was not available in this dataset. Aspiration pneumonia is common in neurosurgical patients with impaired consciousness and neurological dysfunction [35]. Perioperative bleeding is a well-documented risk factor for postoperative infections and patient mortality [25].

In neurosurgery, intraoperative blood loss of up to 350 mL has been found to be an independent predictor of postoperative complications in older patients [36]. However, our study found that intraoperative blood loss does not pose a significant risk for postoperative pneumonia, probably due to a relatively younger patient population. Another possible explanation may be found in the relatively low number of patients with substantial blood loss during surgery.

Patients who received massive intraoperative blood transfusions were more likely to develop postoperative pneumonia. Postoperative blood transfusion was not found to be a strong predictor of postoperative pneumonia. This is probably due to transfusion-related immunomodulation (TRIM). TRIM has been found to increase the number of perioperative and nosocomial infections as well as hospital mortality [37].

However, this study cannot address whether different types of pneumonia may vary in different amounts of preoperative risk factors. Preoperative neurological status is an important component of postoperative pneumonia incidence. For example, some tumors, such as large vestibular schwannomas, can impair cranial nerve function, leading to a higher risk of aspiration pneumonia [26]. Similarly, tumors located near motor pathways can cause patients to become weak and immobile [38]. Postoperative bed rest is associated with the occurrence of postoperative pneumonia, and reducing bed rest is associated with a reduced risk of pneumonia [39]. The present study does not include information on preoperative neurological status or bed rest, which could be helpful in analyzing risk factors for postoperative pneumonia.

Given the lack of data on patients undergoing awake craniotomy, the potential impact of this surgical approach and monitored anesthesia care cannot be assessed within the scope of the current analysis. At the same time, we were unable to quantify the role of postoperative physiotherapy and pneumonia prophylaxis via the TriFlo inspiratory exerciser, which is a standard component of postoperative care in our institution. Another potential limitation of the present result could be the low incidence of postoperative pneumonia, which could underpower the multivariable regression model used in the analysis.

Future research on this topic could result in the establishment of strict criteria for patient selection and risk stratification in the field of neurooncological surgery. This may also assist healthcare professionals, including anesthesiologists and neurosurgeons, in determining the specific risk of postoperative pneumonia and taking this into account during the general preoperative evaluation.

## 5. Conclusions

In summary, this study demonstrates that length of hospital stay and smoking status are risk factors for postoperative pulmonary infections in patients with brain tumors. Nevertheless, the role of prolonged hospitalization as either a cause or consequence remains a subject of debate. In contrast, predictors such as demographic variables (age, sex, blood group) and pre-existing conditions (hypertension, diabetes, cardiovascular disease, renal disease, chronic inflammatory disorders) did not show a significant association with postoperative pneumonia. The findings presented in this study could assist clinical staff in improving the accuracy of risk assessments and perioperative management in patients undergoing brain tumor surgery. In addition, it is expected that these findings will enable the implementation of targeted and effective measures at an early stage, with the aim of preventing postoperative pneumonia and improving clinical outcomes. However, because only a limited number of patients were included, some risk factors may still have influenced the results. Future large-scale, multicenter, high-quality studies are therefore warranted to validate and extend these findings.

## Figures and Tables

**Table 1:** Surgical parameters, demographic data, and pre-existing conditions, as well as their impact on the incidence of pneumonia following surgery.

			Pulmonary Infection		
			No N = 1459 (%) or Mean ± SD	Yes N = 22 (%) or Mean ± SD	*p*-Value
**Demographic data**	Sex	Female Male	762 (52) 697 (48)	7 (32) 15 (68)	0.083
	Age		58 ± 16	55 ± 18	0.5
	BMI		27.3 ± 5.4	28.9 ± 4.1	0.089
	ASA-Classification	I II III–IV	92 (6.7) 868 (60) 488 (33.47)	2 (9.5) 12 (57) 7 (4.35)	0.6
	Smoker	Yes No	382 (27%) 1046 (73%)	1 (4.5%) 21 (95%)	**0.014**
**Surgical** **Parameters**	Duration of the operation [minutes]		199 ± 95	201 ± 74	0.9
	Blood loss [mL]		394 ± 493	424 ± 482	0.8
	Duration of stay [days]		14 ± 8	25 ± 18	**0.011**
**Comorbidities**	Hypertension	Yes No	687 (47%) 772 (53%)	11 (50%) 11 (50%)	0.8
	Diabetes	Type 1/2 No	226 (15%) 1233 (85%)	2 (9.1%) 20 (91%)	0.6
	Liver disease	Yes No	34 (2.3%) 1425 (98%)	1 (4.5%) 21 (95%)	0.4
	Cardiovascular	Yes No	140 (9.6%) 1319 (90%)	2 (9.1%) 21 (90%)	>0.9
	Chronic inflammation	Yes No	105 (7.2%) 1354 (93%)	0 (0%) 22 (100%)	0.4

Body-Mass-Index (BMI), American Society of Anesthesiologists-Classification (ASA-Classification).

**Table 2:** Laboratory parameters’ influence on pneumonia after surgery.

		Pulmonary Infection		
		No (N = 1459) Mean ± SD	Yes (N = 22) Mean ± SD	*p*-Value
**Laboratory** **parameters**	INR	1.00 ± 0.10	0.98 ± 0.04	0.2
	PTT	27.41 ± 3.55	27.40 ± 2.21	0.9
	Platelet count [10*9/L]	269 ± 87	245 ± 54	**0.047**
	C-reactive protein [mg/L]	9 ± 23	11 ± 29	0.7
	Leukocytes [Gpt/L]	9.6 ± 4.0	9.3 ± 4.1	0.8

International Normalized Ratio (INR), partial thromboplastin time (PTT).

**Table 3:** Relevant parameters in multivariate analysis. The other parameters were non-significant.

	OR	95%; CI	*p*-Value
Smoker	0.16	0.01; 0.76	0.071
Duration of stay [7 days]	1.06	1.03; 1.09	0.001
Platelet count [10*10/L]	1.00	0.99; 1.00	0.3

## Data Availability

The datasets obtained and analyzed during the current study are available from the corresponding author on reasonable request.

## Abbreviations

The following abbreviations are used in this manuscript:

ASA-Score	American Society of Anesthesiologists physical status classification system
BMI	Body mass index
PTT	Partial thromboplastin time
TRIM	Transfusion-related immunomodulation
